# Outcomes of robotic, laparoscopic, and open hysterectomy for benign conditions in obese patients

**DOI:** 10.4274/jtgga.2018.0018

**Published:** 2018-06-04

**Authors:** Mostafa A. Borahay, Ömer Lütfi Tapısız, İbrahim Alanbay, Gökhan Sami Kılıç

**Affiliations:** 1 Department of Obstetrics and Gynecology, Johns Hopkins University, Maryland, USA; 2 Department of Obstetrics and Gynecology, University of Texas Medical Branch, Texas, USA; 3 Department of Obstetrics and Gynecology, University of Health Sciences, Etlik Zübeyde Hanım Women’s Diseases Training and Research Hospital, Ankara, Turkey; 4 Department of Obstetrics and Gynecology, University of Health Sciences, Gülhane Training and Research Hospital, Ankara, Turkey

**Keywords:** Hysterectomy, robotic, laparoscopic, open, obese

## Abstract

**Objective:**

To compare outcomes of robotic-assisted (RAH), total laparoscopic hysterectomy (LH), and total abdominal hysterectomy (TAH) for benign conditions in obese patients.

**Material and Methods:**

Retrospective cohort (Class II-2) analysis. All obese patients who underwent RAH, LH or TAH for benign conditions by a single surgeon at the University of Texas Medical Branch between January 2009 and December 2011 were identified and their charts reviewed. The patients’ characteristics, operative data, and post-operative outcomes were collected and statistically analyzed.

**Results:**

A total of 208 patients who underwent RAH (n=51), LH (n=24) or TAH (n=133) were analyzed. There were no significant differences among the groups in demographic characteristics, indications for surgery or pathologic findings. RAH and LH were associated with lower estimated blood loss (EBL) (p<0.001) and shorter length of hospital stay (LOS) (p<0.001) compared with TAH. In addition, RAH and LH had lower intraoperative and early postoperative (≤6 weeks) complications compared with TAH (p=0.002). However, the procedure time was longer in RAH and LH (p<0.001). No significant differences were noted among the groups for late post-operative complications (after 6 weeks) or unscheduled post-operative visits.

**Conclusion:**

Minimally invasive hysterectomy appears to be safe in obese patients with the advantages of less EBL, fewer intraoperative complications, and shorter LOS.

## Introduction

Obesity is defined by the World Health Organization as having a body mass index (BMI) ≥30 kg/m^2^ (1). The prevalence of obesity among adults in the United States of America stayed around 15% from 1960 to 1980 before rapidly accelerating from 13.4% in 1980 to 35.7% in 2010 (2) and is projected to reach 42% by 2030 (3). Obesity is a well-known risk factor for medical problems (4) and surgical outcomes including hysterectomy (5,6,7,8).

Laparoscopic hysterectomy (LH) was first described in 1989 (9) and has been demonstrated to be safe and feasible (10). Its advantages include less blood loss, less post-operative pain, shorter hospital stay, faster recovery and better cosmetic outcome. More recently, the United States Food and Drug Administration approved the da Vinci^®^ Surgical System (Intuitive Surgical Inc., Sunnyvale, CA) for hysterectomy in 2005. Thereafter, robotic hysterectomy has been reported to have several enhancements including improved dexterity with EndoWrist movements and 3D visualization (11,12,13). These enhancements are critical, especially in complex cases where extensive dissection is required (14). For these advantages, laparoscopic and robotic hysterectomy have been gaining momentum (13,15,16).

With the current epidemic-like status of obesity (17), and the medical risks it poses (4), it has also been demonstrated to pose a substantial surgical risk (5). In fact, it has been reported that obesity is associated with increased intra- and post-operative complications including bleeding and infections in patients undergoing hysterectomy (18). Whether obesity-related complications depend on the approach of hysterectomy for benign conditions is not clearly determined at the present time. With the growing adoption of robotic and LH, there is a growing need for more evidence about the safety and outcomes of laparoscopic and robotic hysterectomy in the obese. Although current evidence suggests that LH is associated with fewer complications than abdominal hysterectomy in obese patients (5), most of these studies include patients with cancer (19,20,21,22). There is a need for data exclusively from benign cases because patients with malignancy have a different outcome. The objective of this study was to analyze the outcomes of robotic and LH for benign conditions in obese patients in comparison to open approaches.

## Material and Methods

This retrospective cohort study (Class II-2) was approved by the Institutional Review Board. Informed consents from patients were not required because it is retrospective chart review study. All obese patients who underwent robotic assisted hysterectomy (RAH), total LH or total abdominal hysterectomy (TAH) by a single surgeon from January 1^st^, 2009, to December 31^st^, 2011, were identified. Obesity was defined as having a BMI ≥30 kg/m^2^ (1). All patients were thoroughly counseled about the risks and benefits and they chose the route of surgery. Patients with pre-operative diagnosis of gynecologic cancer were excluded.

All patients received standard antibiotics and thromboembolic prophylaxis according to the American College of Obstetricians and Gynecologists guidelines (23,24). The RAHs and LHs performed in this study were American Association of Gynecologic Laparoscopists type IVE, defined as total laparoscopic removal of the uterus and cervix including vaginal cuff closure (25). Patients undergoing RAH and LH were placed in the dorsal lithotomy position with Allen stirrups (Allen Medical System, Acton, MA). In this study, we followed the “Strengthening the Reporting of Observational Studies in Epidemiology guidelines (26).

Electronic medical records were reviewed and de-identified data were extracted and double-checked for missing values. The following pre-operative characteristics were obtained: age, race, gravidity, parity, BMI, prior pelvic/abdominal surgery including peritoneal entry, smoking status, medical problems, and indications for surgery. In addition, we collected the following operative data: procedure time (skin-to-skin), estimated blood loss (EBL), concomitant procedures, conversion to open route, specimen morcellation, intraoperative complications (defined as bleeding ≥500 mL; injury to bladder, ureter or bowel and significant ventilation problems), and transfusion (intra-operative and post-operative within 6 weeks). Finally, we included the following peri-operative characteristics: length of hospital stay (LOS), uterine weight, final pathologic diagnosis, and hospital readmission (within 6 weeks).

All patients were followed for one year. Post-operative complications were defined as: fever (body temperature ≥38 °C on 2 consecutive occasions at least 6 hours apart, excluding the first 24 hours); urinary tract infection; urinary retention (without a concomitant urinary incontinence procedure); pelvic hematoma or abscess; genitourinary fistulas; cuff dehiscence; positional nerve injuries; and port-site, cardiopulmonary, gastrointestinal (ileus or bowel obstruction) and ophthalmologic complications were retrieved and subdivided into early (within 6 weeks) or late (from 6 weeks to 1 year). Patients were asked if they had presented to other hospitals and data were obtained whenever applicable.

### Statistical analysis

The first step in the data analysis was to double-check data for missing values. Means and standard deviations were calculated for continuous variables. Then, bivariate relationships were assessed using frequency cross-tabulation for categorical variables. One-way analysis of variance with Bonferroni post hoc analysis whenever applicable, was used for continuous variables, and the chi-square test and Fisher’s exact test were used for categorical variables when appropriate. Data were analyzed using Statistical Analysis Software (SAS), v. 9.2 (SAS Institute, Cary, NC). In all instances, a p value <0.05 was considered statistically significant. 

## Results

A total of 208 consecutive hysterectomy cases were analyzed including RAH (n=51), LH (n=24), and TAH (n=133). Characteristics of the study population are summarized in Table 1. As shown, differences among groups in BMI and other characteristics including age, race, parity, history of medical problems, smoking status and indications for surgery were not significant. However, the TAH group had more prior abdominal/pelvic surgeries (p<0.001). 

Next, we analyzed operative data and post-operative outcomes (Table 2). As shown, there were no significant differences among the groups in oophorectomy, concomitant procedures, conversion rate (only RAH and LH), or morcellation (only RAH and LH). There were three conversions in the RAH group for bleeding, PK malfunction (converted to laparoscopy), and a minilaparotomy for specimen retrieval. In comparison, there were 2 conversions in the LH group for a patient who could not tolerate the Trendelenburg position and another for instrument malfunction. Intra-operative complications were higher in the TAH and LH groups compared with the RAH group (p=0.002). The vast majority of these complications were excessive intra-operative blood loss (n=2, n=3, and n=32 in the RAH, LH, and TAH groups, respectively).

In addition, we found that RAH and LH were associated with less EBL compared with TAH (p<0.001) with post hoc analysis showing significantly less EBL in RAH compared with LH (p=0.019). Furthermore, we found that TAH was associated with more blood transfusions compared with RAH and LH; however, the difference was not statistically different (p=0.052). 

On further analysis, we found that RAH and LH had significantly longer procedure times compared with TAH (p<0.001) with Bonferroni post hoc analysis showing no significant difference between RAH and LH (p=0.056). The LOS was significantly shorter in the RAH and LH groups (p<0.001). In addition, early post-operative complications (≤6 weeks) were lower in the LH group compared with RAH and TAH groups (p=0.002). However, late post-operative complications (between 6 weeks and up to 1 year after surgery) were not significantly different among the groups (p=0.113). Finally, there was no significant difference between groups in terms of final pathologic diagnosis (p=0.085). However, the uterine weight was highest in the TAH group (p<0.001), with Bonferroni post-hoc analysis showing significant differences between the RAH and LH groups (p=0.015). 

## Discussion

The results of this study demonstrate that RAH and LH in obese patients are associated with less EBL, fewer intraoperative and early postoperative complications, less perioperative blood transfusion, and shorter LOS, although they require longer operating times compared with TAH. Moreover, EBL, LOS, and perioperative blood transfusion were noted to be less in the RAH group when compared with LH. 

The findings in this study are in line with other studies. Gali et al. (27) found that RAH was associated with shorter hospital stays, and fewer infectious complications compared with TAH. Geppert et al. (19) also compared RAH with TAH in obese patients. The authors reported similar results and concluded that RAH was feasible but required training and special expertise. Another study by Eddib et al. (28) examined the impact of BMI on surgical outcomes of RAH. They concluded that procedure time was longer in morbidly obese patients; however, obesity had no impact on other outcomes. In contrast to this study, Nawfal et al. (29) reported no association between BMI and duration of surgery and similarly concluded that RAH might be a better approach to hysterectomy in obese and morbidly obese patients. Boggess et al. (30) compared outcomes in RAH, LH, and TAH in patients with endometrial cancer. The mean BMIs were 32.9, 29.0, and 34.7, respectively, and the results favored a robotic approach in terms of blood loss, hospital stay, and post-operative complications. In another study that compared RAH and TAH in obese women who underwent surgical staging for endometrial cancer, similar results and conclusions were reported (20).   

This study has certain strengths. First, all procedures were performed by a single surgeon, eliminating potential confounding factors when analyzing cases performed by multiple surgeons. In addition, this study exclusively includes procedures performed for benign indications. This is in contrast to other studies in which patients with cancer were included in the analysis along with benign cases, which affects the validity of the outcomes. However, the study also has some limitations. First, the study design is a retrospective cohort analysis. We believe that this may have affected the results, potentially due to selection bias. Therefore, prospective randomized trials are needed to overcome this limitation. In addition, the sample size, especially of the LH group, was relative small and the study groups were not equal in size. Consequently, larger studies are needed to confirm the findings in this study. Finally, this study was performed in a teaching institution where residents participated in most cases. This needs to be considered when analyzing the study results, especially procedure time. However, as residents participated equally in the study groups, we do not think that this factor had an impact on the study conclusions.

There is a clear need to further investigate different clinical and financial aspects of minimally invasive hysterectomy in obese patients because we currently counsel obese patients based primarily on data from the general population. The initial evidence suggests that minimally invasive hysterectomy is safe and feasible in obese patients. For example, Gali et al. (27) examined the effects of the steep Trendelenburg position on cardiopulmonary function in obese patients. The authors found that although higher inspiratory pressures were needed in RAH compared with TAH, cardiopulmonary complications were not significantly different. However, several other variables and outcomes were not examined. For example, despite evidence that intraocular pressure goes up with the steep Trendelenburg position during minimally invasive gynecologic surgery (31), the magnitude of this effect has not been evaluated in obese patients. Also, although outpatient robotic hysterectomy was demonstrated to be safe and associated with financial savings (32,33,34), its safety and feasibility has not yet been evaluated in the obese patient subset. Similarly, costs of robotic gynecologic surgery in benign cases were analyzed with strategies for efficiency (35,36), but there is paucity of the effect of BMI on cost in benign robotic hysterectomy. In addition, because the incidence of occult cancer discovered after minimally invasive gynecologic surgery has been examined (37), there is a need to examine it in the obese patient population. All these clinical characteristics are important for the accurate counseling of obese patients. Also, with the current evidence of disparities in the use of LH (38), it is important to determine if it is adequately used for the obese patient population. Finally, there is a clear need to take important clinical variables such as BMI into account when designing and using simulators, which appear helpful in minimally invasive gynecologic surgical training (39,40).

In conclusion, our study demonstrates that in spite of a longer procedure time, robotic and laparoscopic hysterectomies are feasible, safe, and provide shorter hospital stays and less blood loss in the obese patient population. Finally, larger, prospective, randomized studies that also evaluate other clinical and financial outcomes are recommended.

## Figures and Tables

**Table 1:**
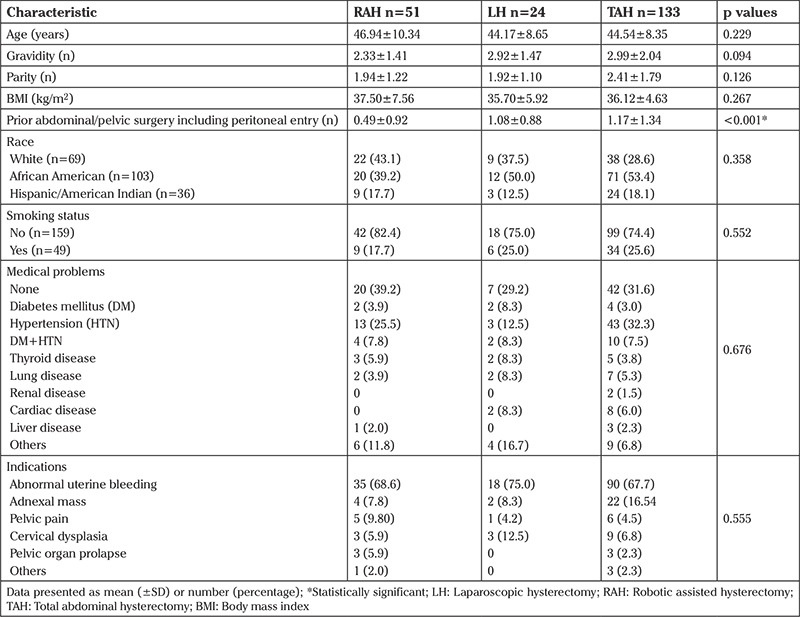
Characteristics of study population

**Table 2:**
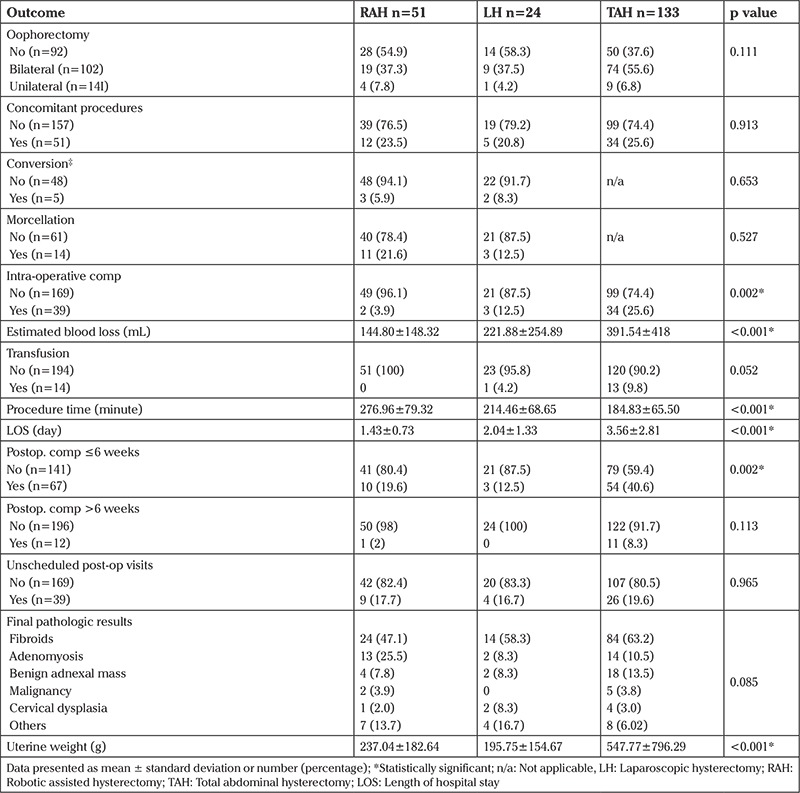
Operative data and post-operative outcomes
